# A priori sample size determination and power analysis in metabolic phenotyping and integrative metabolomics: an application framework based on a systematic review of literature

**DOI:** 10.1007/s11306-026-02464-y

**Published:** 2026-06-19

**Authors:** Nicola Luigi Bragazzi, Sara Dobani, José Fernando Rinaldi de Alvarenga, Cristiana Mignogna, Daniele Del Rio, Pedro Mena

**Affiliations:** 1 Human Nutrition Unit, Department of Food and Drug, Medical School, University of Parma, Building C, Via Volturno 39, 43125 Parma, Italy; 2 Microbiome Research Hub, University of Parma, Parco Area delle Scienze 27/A, 43124 Parma, Italy

**Keywords:** Metabotypes, Multi-omics, Sample size estimation, Power calculation

## Abstract

In biomedical research, predetermining the appropriate number of samples is essential to ensure the validity of findings, optimize resource allocation, and support meaningful scientific discovery. Accurate sample size estimation is particularly critical in complex study designs, such as those found in metabolomics, including metabolic phenotyping and integrative metabolomics. However, this task remains challenging due to the high dimensionality and variability inherent in metabolomics data. In recent years, efforts have been made to devise techniques and applications that could assist in designing and implementing metabolomics studies. Despite these efforts, a comprehensive evaluation of existing approaches is lacking, limiting the guidance available to researchers and potentially hindering progress in the field. To address this gap, a systematic literature review was conducted, mining two major scholarly databases (Scopus and MEDLINE *via* PubMed) and identifying twenty relevant studies. This review aims to provide an overview of the currently available methodologies for conducting a priori sample size calculations and power analyses in metabolomics, while also highlighting ongoing challenges and outlining directions for future research.

**Supplementary Information:**

The online version contains supplementary material available at https://doi.org/10.1007/s11306-026-02464-y.

## Introduction

The detection of relevant and clinically meaningful biomedical effects relies on accurately determining the number of samples and observations before formally commencing the study itself (Pourhoseingholi et al., 2013). Achieving a sufficiently powered sample size can impact several aspects of the research process, from the formulation of the research question to its implementation, including experimental design, generated costs, time and resource constraints, ethical considerations, and potential novel insights and discoveries (Emwas et al., 2025; Serdar et al., 2021). Research outcomes are the trade-off between the influence of chance, in terms of random variation and variability that may potentially undermine scientific validity, and reproducibility and efficiency, in terms of time and resources allocated (Howard, 2002). Statistically underpowered sample sizes provide researchers not only with a reduced chance of detecting true effects, but also with a limited probability that a significant result may reflect an actual outcome. Besides statistical implications, other logistical and organizational factors impacted by the selected sample size, such as laboratory supplies, equipment, and personnel, should be considered (Pourhoseingholi et al., 2013; Serdar et al., 2021).

In the last few decades, challenges in determining sample size have increased along with the complexity of study designs, which often involve repeated measurements, multiple treatment groups, cluster randomization, or factorial designs. They require employment of advanced statistical methods, while adding uncertainty in sample size estimation (Baio et al., 2015; Rutterford, Copas et al., 2015; Rutterford, Taljaard, et al. 2015). This is especially the case of metabolomics studies, including metabolic phenotyping and metabotyping investigations, which refer to comprehensive analysis of metabolites in biological samples to characterize the metabolic profile of a given organism and the classification of individuals or samples into distinct metabolic profiles, respectively (Hillesheim et al., 2023; Noerman et al., 2020). In these studies, sample size determination remains a complex and critical step, due to the high chemical diversity of metabolites and the incredible wealth of high-dimensional data, which are dense, intercorrelated, and have been gathered and generated through high-throughput methodologies and top-down hypothesis-free approaches (Bartel et al., 2013; Klupczyńska et al., 2015). Moreover, besides the multivariate and multiparametric nature of metabolomics data, the catalog of human metabolome is still far from complete, and it is not infrequent to detect unknown metabolites (Dührkop et al., 2021; Wishart et al., 2022), which could benefit from reverse metabolomics application (Gentry et al., 2024).

The scarcity of pilot studies in metabolomics, which are crucial for providing preliminary data that can inform more accurate sample size calculations, further complicates the determination of sample size. Additionally, despite recent advancements, the accuracy, precision, and reproducibility of metabolomic data are highly dependent on the quality of samples and methodologies used, and variability in these aspects can lead to significant uncertainties in study outcomes (Cochran et al., 2024). Moreover, the integration of multi-omics data, which combines metabolomics with genomics, transcriptomics, proteomics, microbiomics, and other related fields, introduces additional layers of complexity in sample size determination, which requires specific considerations and adaptations of tools for this experimental design (Tarazona et al., 2020). The issue of biological variability cannot be overstated. Individual differences in metabolism, resulting from genetics, gut microbiota composition, lifestyle, diet, and environmental exposures, add another level of complexity to metabolomic studies (Favari et al., 2024; Martínez-González et al., 2025; Narduzzi et al., 2022). This inherent variability underscores the need for larger sample sizes to ensure that the study findings are both robust and generalizable across diverse populations.

When dealing with analytical and computational workflows in metabolomics, lack of standardisation is evident, particularly across pre-analytical and analytical phases. Variations in sample type, collection, storage and handling can introduce systematic biases (González-Domínguez et al., 2020), while instruments and batch effects further contribute to metabolite variability (Shi et al., 2022; Stancliffe et al., 2022). Inter-laboratory comparisons have shown that even if similar sample preparations are used, certain metabolite annotations and relative quantifications are not reproducible, undermining the transferability of variance estimates used for power analyses (Lin et al., 2020). A further challenge arises from the limited knowledge of population-level variability in metabolite concentrations. Demographic and environmental factors strongly influence baseline metabolite levels (Stancliffe et al., 2022; Tolstikov et al., 2020). Moreover, from a computational perspective, different statistical strategies, ranging from pre- to post-processing methods, are applied, potentially altering metabolite levels (Blaise et al., 2021). These inconsistencies hinder cross-study comparability and complicate the estimation of variability and effect sizes for robust power analysis in metabolomics.

**Fig. 1:**
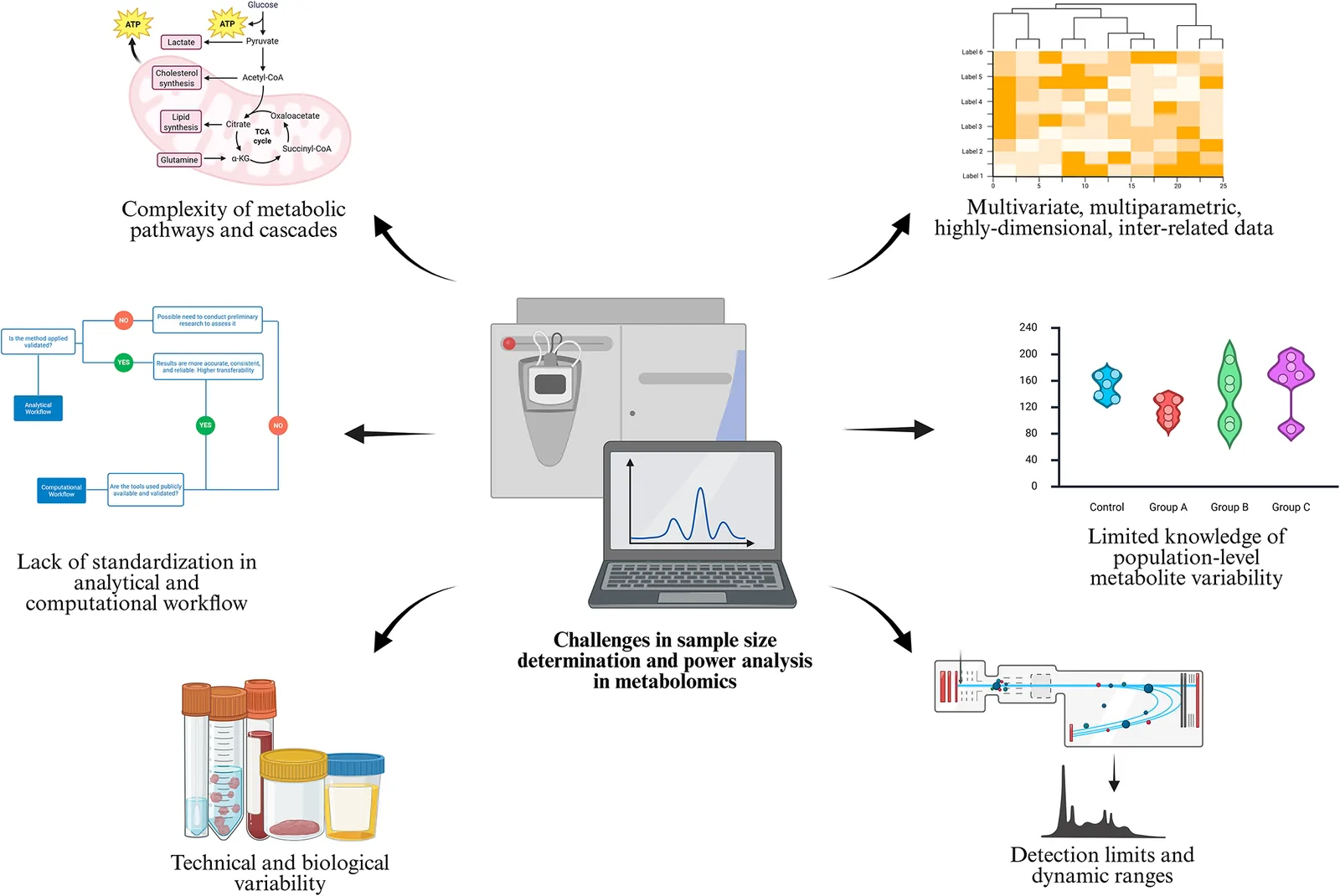
Overview of the major challenges that characterize metabolomics data. Created in https://BioRender.com

Due to these challenges (Fig. 1), there is no actual consensus about the best method to perform an a priori sample size calculation and power analysis in the field of metabolomics, especially when pilot studies are lacking (Johnson & Gonzalez, 2012) and when there are not a priori known target metabolites (Dührkop et al., 2021; Wishart et al., 2022). Their concentration is in a broad dynamic range and often dependent on the methodology chosen for extracting, detecting, identifying, measuring, handling, and processing the metabolites (Clark et al., 2003; Pinto et al., 2014; Worley & Powers, 2012), as well as on the type of samples collected, analytical assay chosen, and platform used, which further complicate standardization (Stevens et al., 2019; Xiao et al., 2014). Some metabolites are shared among several metabolic pathways and cascades (Cuperlovic-Culf, 2018), making them particularly difficult targets for statistical analysis. These complexities collectively hinder the development of universally applicable strategies for determining the proper sample size and performing power calculation analyses in metabolomics (Johnson & Gonzalez, 2012; Worley & Powers, 2012).

Tools for making these computations based on standard univariate techniques exist, whereas, in multivariate scenarios, there are only a few extensions of the classical Student’s t-test, such as the multivariate Hotelling’s T^2^ statistics, or the multivariate analysis of variance (MANOVA) (Johnson & Gonzalez, 2012; Worley & Powers, 2012). However, these solutions are unfeasible in the field of metabolomics, due to the singularity of the sample covariance matrix and the subsequent lack of existence of its inverse (Johnson & Gonzalez, 2012; Worley & Powers, 2012). This is further challenged by the widespread use of unsupervised techniques, which may result in unstable and non-reproducible results, and consequently, a lack of predictive power (Anwardeen et al., 2023). Moreover, very few studies have conducted metabolic phenotyping with contrasting findings (Hillesheim & Brennan, 2020; Riedl et al., 2017).

Considering the fundamental importance of a priori sample size calculation and power analysis in metabolomics studies, a systematic literature review was conducted to provide an overview of the currently available methodologies for these analyses. Particular attention was paid to tools considering metabolic phenotyping and integrative metabolomics. This review aims to provide researchers with application guidance on the use of these methods, identify existing gaps in knowledge, and present trends and new directions in the field.

## Materials and methods

### Study protocol and finding reports

An a priori study protocol was developed and is reported in Supplementary Methods. The protocol was not submitted to the “International Prospective Register of Systematic Reviews” (PROSPERO) for registration, as it has a major methodological focus rather than a clinical focus.

The present study was conducted in accordance with the “Preferred Reporting Items for Systematic Reviews and Meta-Analysis” (PRISMA) checklist and guidelines (Page et al., 2021).

### Literature search

Two major scholarly electronic databases, namely MEDLINE, *via* its PubMed interface, and Scopus, were searched in May 2024 and updated in May 2025 by two researchers (NLB, SD). A search string consisting of keywords such as metabolomics, sample size estimation, and power analysis, properly connected by Boolean operators, was formulated as described in Supplementary Table 1. “Medical Subject Headings” (MeSH) terms and wild-card options (i.e., truncated words) were used, when relevant. Extensive cross-referencing was employed, and target journals in the field of metabolomics were manually searched.

### Inclusion and exclusion criteria

Inclusion and exclusion criteria were formulated a priori, along with the study protocol, before formally commencing our investigation, following an initial familiarization with the existing scholarly literature on the topic.

Studies of any design (full research papers, technical notes, software papers) providing methodological insights on sample size estimation and power analysis in the field of metabolomics were deemed eligible, as described in the Supplementary Methods. Exclusively theoretical papers, not providing applications, software, or not carrying out validation of their methodologies on metabolomics datasets, were excluded. Reviews, if any, even though ineligible, were scanned to increase the chance of including relevant studies in the current work.

### Data extraction and synthesis

An *ad hoc* Excel spreadsheet was created for data extraction. Researchers extracted the following information: study authors, name of the application, programming language used, operating systems on which the application can run, number and type of parameters that have to be inputted by the user, type of statistical analysis implemented, and type of metabolomics datasets (artificial/simulated or real-life/experimental) used for the validation.

Data extracted were synthesized in a narrative fashion, also using tables and charts.

### Study classification framework

To improve conceptual clarity and facilitate comparison across included studies, they were classified according to the primary statistical strategy used for sample size determination and power analysis. Classification was inductively conducted after full-text assessment and was based on the dominant inferential framework and data requirements of each study. Specifically, we evaluated whether the approach relied on (i) synthetic data generation or resampling procedures, (ii) empirical effect-size and variance distributions derived from observed datasets, or (iii) classical parametric analytical formulae. Accordingly, studies were grouped into three broad methodological classes: simulation-based approaches; empirical distribution–based analytical approaches; and classical analytical approaches. This structured categorization informed the organization and comparative interpretation of the results.

## Results and discussion

### Literature search

**Fig. 2:**
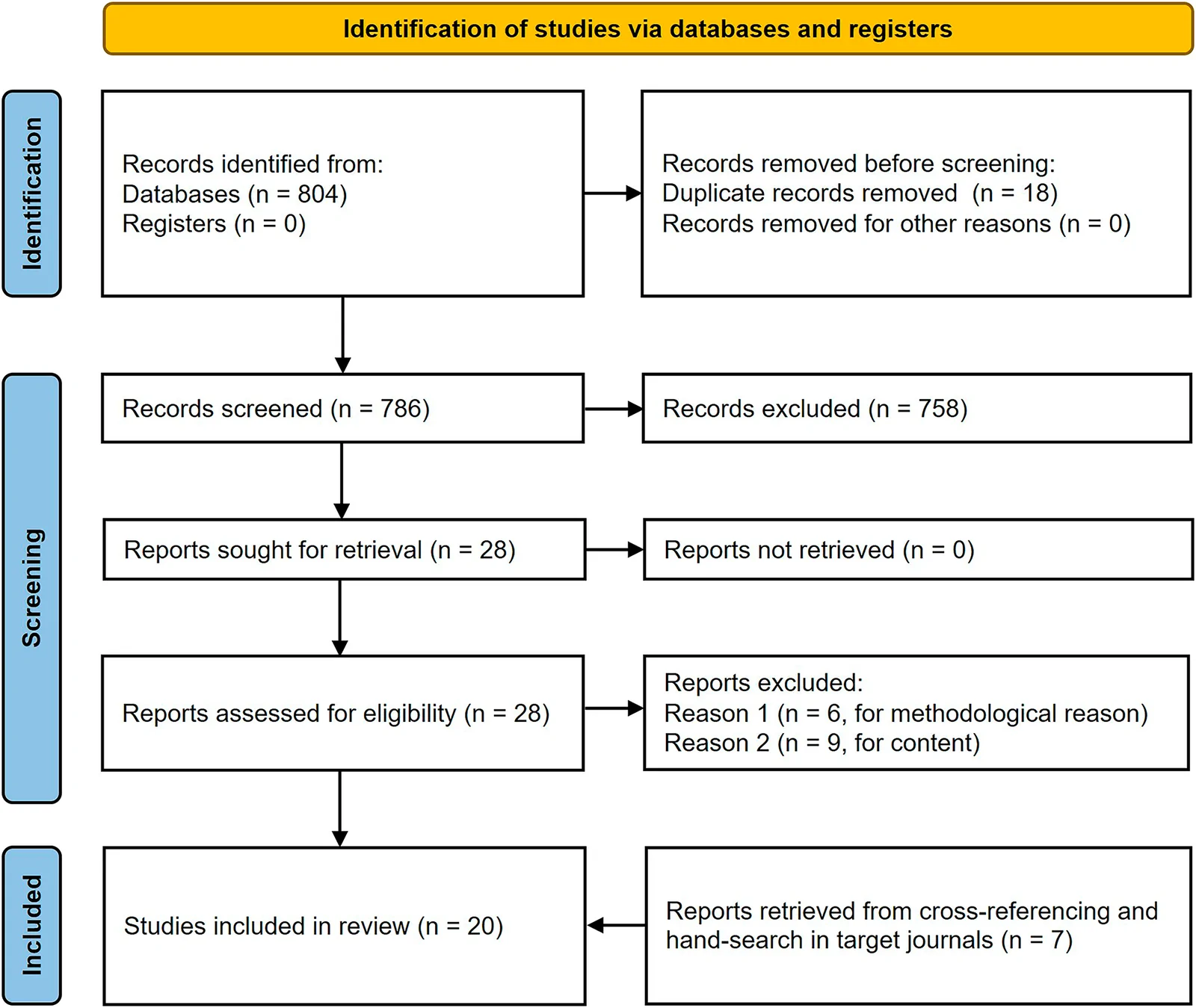
Pictorial diagram of the search strategy adopted in the present systematic review

The initial literature search yielded 804 items. After removing duplicates, 786 articles were screened: based on title and abstract, 758 items were removed. Twenty-eight studies were retrieved in full text and carefully assessed. Out of these, fifteen studies (Camacho et al., 2023; Chi et al., 2014; Dufault et al., 2023; Duisters et al., 2020; Fernández-Albert et al., 2014; Hendriks et al., 2011; Jo et al., 2018; Jo & Park, 2017; Ma et al., 2024; Narita et al., 2021; Saccenti & Timmerman, 2016; Taylor et al., 2013; Vinaixa et al., 2012; Wheelock & Wheelock, 2013; Yu & Huan, 2022) were excluded with reasons: six because they were merely methodological, nine lacked sufficient or relevant detail to meet the inclusion criteria. Finally, twenty studies, including seven papers retrieved from cross-referencing and search in field specific journals (Acharjee et al., 2020; Billoir et al., 2015; Blaise, 2013; Blaise et al., 2016, 2021; Chong et al., 2018, 2019; Chong & Xia, 2020; Li et al., 2020; Liang et al., 2020; Nyamundanda et al., 2013; Syed et al., 2021; Van Iterson et al., 2009; Iterson et al., 2013; Wanichthanarak et al., 2017; Wen et al., 2017; Wen, 2020; Xia et al., 2013, 2015; Xia & Wishart, 2016) were retained and overviewed in the present systematic review, as pictorially shown in Fig. 2. Selected studies were published between 2009 and 2021. Each of the included studies provided practical operative instructions. The main characteristics of the nine applications and methodologies implemented in the selected studies are listed in Table 1.

**Table 1:** Main characteristics of the studies included in the present systematic review

Application name	Programming language	Operating systems	Pilot study required	Study design	Pre-processing	Main parameters inputted by user	Tests	Statistical approach	References
MetaboAnalyst	R (MetaboAnalystR) with a web-based interface built using R/Shiny plus HTML/JavaScript/CSS	Web-based (works on any browser: Windows, macOS, Linux)	No	Two-group (unpaired or paired) and multi-group designs	Sample normalization (none, sample-specific, by weight or volume, by sum, by median, by reference sample/PQN, by pooled sample from group/group PQN, by reference feature, quantile), data transformation (none, log transformation, square root transformation, cube root transformation), data scaling (none, mean centering, auto-scaling by the standard deviation of each variable, Pareto scaling, range scaling), filtering, and missing-value imputation	Choice of normalization, transformation, scaling; statistical test type; effect size; significance level (α); desired power; sample size grid	Univariate tests (t-test, ANOVA, nonparametric tests); multivariate analyses (PCA, PLS-DA, sPLS-DA, OPLS-DA, clustering); machine learning (including RF); multiple-testing correction (Benjamini–Hochberg FDR)	Classical analytical	Chong et al. (2018, 2019), Chong and Xia (2020), Xia et al. (2013, 2015), Xia and Wishart (2016), Van Iterson et al. (2009), and Iterson et al. (2013)
MetSizeR	R, available also with a GUI interface	Platform-independent, mainly Windows	No	Two-group designs; can also handle covariates (PPCCA) and longitudinal designs (DPPCA) as well as other study designs based on statistical models	Required, as the application can deal with a maximum of 375 variables; if needed, it must be performed by the user	Number of bins/metabolites, expected proportion of significant bins/metabolites, target FDR, minimum sample size per group, statistical model, number of covariates to be included	Two-sample t-statistics, permutation statistics, PPCA, PPCCA, DPPCA; FDR control *via* Benjamini–Hochberg correction	Simulation-based	Nyamundanda et al. (2013)
DSD	MATLAB, GNU Octave *via* command line	Cross-platform (Linux, Windows, and MAC OS X)	Yes	Two-group designs (can be adapted to multi-group *via* ANOVA)	The application can deal with pre-processed or raw data through SRV to identify metabolic variables; kernel smoothing density or log-normal estimation; optional noise-removing filter	SRV parameters (singlet peak base width, bucketing resolution, correlation threshold), significance threshold (α), simulation size (*n* = 20–400), effect definition (biomarker discovery vs. metabolic exploration)	One-way ANOVA; FDR control *via* Benjamini–Yekutieli correction; ROC/resampling under the null hypothesis, cross and independent model validation; OPLS on SRV clusters	Simulation-based	Billoir et al. (2015), Blaise (2013), and Blaise et al. (2016, 2021)
PowerTool	R with Shiny web interface	Linux, Windows, and MAC OS X	Yes	Supports both binary classification and regression outcomes for translational metabolomics studies	If needed, it must be performed by the user	Outcome variable (regression or two-group classification), range of sample sizes/sample size grid, number of iterations *per* variable, selection stringency (≥ 5/100 or ≥ 90/100 iterations), correlation-grouping threshold, and threshold for significance	ANOVA for binary outcomes, linear regression for continuous outcomes, effect size estimation (Cohen’s d for classification, Pearson correlation for regression), simulation *via* multivariate log-normal with empirical correlation structure, nested cross-validation, RF (Boruta, permutation-based feature selection with and without correction, backward elimination-based feature selection/ recursive feature elimination), performance/confusion matrix; FDR control *via* Benjamini–Hochberg for permutation testing	Simulation-based	Acharjee et al. (2020)
Ssizer	R (Shiny web interface using multiple R packages and the Bioconductor SSPA package)	Web-based, implemented on CentOS Linux, accessible through any web browser on Linux, Windows, and MAC OS X	Yes	Two-group designsTwo-groupsdesigns	Data transformation (none, log transformation, cube root transformation), normalization (none, auto-scaling, contrast, cubic splines, cyclic LOESS, EigenMS, linear baseline, level scaling, MSTUS, power scaling, Pareto scaling, Power scaling, PQN, quantile, range scaling, vast scaling, variance stabilizing norm), missing value input, and filtering (none, mean, SD)	Number of samples of the two groups, pre-processing parameters	Descriptive statistics, data visualization, Students’ t-test, PCA, AUC/ROC, SVM, RF, DL-DA, PLS-DA, and OPLS-DA, CV including 5-fold CV	Simulation-based	Li et al. (2020)
Metabox	R (with HTML, JavaScript, CSS GUI *via* OpenCPU, D3.js, Cytoscape.js)	Installable as R package or web-based (running on any browser, *via* Linux, Windows, and MAC OS X)	No	Two or multiple paired or independent groups	Data transformation (logarithm, power), normalization (sum, LOESS, batch-median, sample metadata-based), scaling (Pareto, range, autoscaling), outlier detection, missing value handling	Meta-data, features and quantified data (e.g., metabolite intensities, expression values)	Power analysis provided at the entity level for several tests, including Student’s t-test, Welch’s t-test, Mann-Whitney U test, Kruskal-Wallis rank-sum test, ANOVA, Welch ANOVA, repeated ANOVA, Friedman test, two-way ANOVA, two-way repeated ANOVA, mixed ANOVA, robust ANOVA; other statistical analyses include bootstrapping; PCA; network construction (biological, correlation, chemical similarity); functional class scoring; overrepresentation; word cloud generation; FDR control *via* Benjamini-Hochberg	Classical analytical	Wanichthanarak et al. (2017)
IP4M	Java, Perl, R, GUI with Eclipse RCP	Windows, Ubuntu/Linux, and macOS	No	Two/more groups	The application can deal with pre-processed or raw data; extensive pre-processing including peak picking, deconvolution, and annotation (metaMS/XCMS/CAMERA; eRah for GC–MS), isotope filtering, pseudospectra, alignment, missing-value imputation (minimum, KNN, qrilc), transformation (log, z-score), normalization (total signal, internal standard, QC-based), outlier replacement, ratio-variable generation (KEGG-derived reaction pairs)	Expected effect size, significance threshold (for Student’s t-test, either paired or unpaired, ANOVA, correlation, comparison of proportions, GLM) for power analysis; data-processing/normalization choices for the full workflow	Univariate (Student’s t-test, Wilcoxon test, ANOVA, and Kruskal-Wallis rank-sum test) and multivariate (PCA, PLS-DA, OPLS-DA, SVM, and RF) statistics, integrated methods (biosigner and Boruta), regression, GLM, correlation and distance analysis, clustering, ROC analysis, pathway and enrichment analysis, corrections for multiple testing (Bonferroni, Holm and FDR)	Classical analytical	Liang et al. (2020)
metaX	R, with a web-based GUI or in the form of command line functions, Java; power and sample size analysis function is based on the Bioconductor package SSPA	Platform-independent (Linux, Windows, and MAC OS X)	No	Two group comparison	Peak picking and annotation (*via* XCMS and CAMERA) and extensive pre-processing, including data quality assessment, missing-value imputation (KNN, BPCA, SVD, RF), transformations (logarithm/generalized logarithm/cube root transformations), outlier removal (Hotelling T^2^ ellipse), normalization (Total Sum, PQN, VSN, Quantile, QC-RSC, SVR, ComBat), scaling (Pareto, Vast, Range, Auto, Level)	Peak table or mzXML input, filtering thresholds, imputation method, normalization/scaling/transformation choices, CV threshold, statistical test options, SSPA settings for power/sample size curves	Univariate statistics (Student’s t-test, Mann-Whitney U test, ROC), multivariate statistics (cluster analysis, PCA, PLS-DA, OPLS-DA), correlation and differential correlation network analyses, metabolite pathway analysis, metabolite selection (RF, SVM), FDR control *via* Benjamini-Hochberg	Empirical distribution–based	Wen (2020) and Wen et al. (2017)
MOPower	R, Python (R–Shiny interface)	Cross-platform (Linux, Windows, and MAC OS X)	No	Case–control and time-to-event (survival) designs; supports both single- and multi-omics study structures	Not required (data simulated internally)	Number of simulations, initial sample size/sample size grid, case–control ratio/treatment allocation ratio or survival model parameters, treatment effect size, number of omics features, fold change between groups, integration model choice (optional), and type I error rate	Integrative multi-omics analysis (jRM, MA, MOFA, jNMF, SNF, GLM and Cox Path with L1 penalty, exact test, NB generalized log-linear regression model); FDR control *via* Benjamini-Hochberg and Bonferroni	Simulation-based	Syed et al. (2021)

*ANOVA* analysis of Variance, *DL-DA* Diagonal Linear Discriminant Analysis, *DPPCA* Dynamic Probabilistic Principal Component Analysis, *FDR* False Discovery Rate, *GLM* Generalized Linear Modeling, *jNMF* Joint Non-negative Matrix Factorization, *jRM* Joint Regression Modeling, *MA* Mediation Analysis, *MANOVA* Multivariate Analysis of Variance, *MOFA* Multi-omics Factor Analysis, *NB* Negative Binomial, *OPLS-DA* Orthogonal Partial Least Squares Discriminant Analysis, *PCA* Principal Component Analysis, *PLS-DA* Partial Least Squares Discriminant Analysis, *PPCA* Probabilistic Principal Component Analysis, *PPCCA* Probabilistic Principal Components and Covariates Analysis, *PQN* Probabilistic Quotient Normalization, *RF* Random Forest, *ROC* Receiver Operating Characteristic, *SNF* Similarity Network Fusion, *SRV* Statistical Recoupling of Variables, *SVM* Support Vector Machine

### Overview of the studies included

Xia and colleagues are the developers of the application MetaboAnalyst (Chong et al. 2018, 2019; Chong & Xia, 2020; Xia et al., 2013, 2015; Xia & Wishart, 2016), a comprehensive platform streamlining metabolomics data analysis available both as a user-friendly, web-based interface (https://www.metaboanalyst.ca/) and an R package (MetaboAnalystR). They have developed an online module that enables power analysis and sample size estimation, whereby users specify the expected effect size, variance estimates, significance level, and desired power.

Wen (2020) and Wen et al. (2017) devised metaX, an application written in R, with a web-based graphical user interface (GUI) or in the form of command line functions, running on Linux, Windows, and MAC OS X (http://metax.genomics.cn/). A set of metabolomics biomarkers associated with an outcome variable is inputted by the user. The application can handle data quality assessment, missing value input, data normalization, and conduct power analyses for univariate and multivariate statistics. The sample size analysis function is based on the Bioconductor package SSPA, like MetaboAnalyst.

Nyamundanda et al. (2013) have developed a package in the R environment, MetSizeR (https://cran.r-project.org/web/packages/MetSizeR/), which enables a priori sample size calculation and power analysis by means of an analysis-based approach. Due to the limited number of pilot studies in metabolomics, this tool provides the option to create sample sizes with and without prior data. This analysis can be a probabilistic principal component analysis (PPCA), a probabilistic principal components and covariates analysis (PPCCA), or a dynamic PPCA (DPPCA). The researcher can set different input values, such as the type of metabolomic technique employed (targeted or untargeted), the level of false discovery rate (FDR) according to the Benjamini–Hochberg correction, the number of expected metabolites or bins, and the anticipated proportion of significant metabolomic markers, the number of expected minimal samples *per* group or cluster, their ratio, and the number of numeric and categorical variables (including their levels). This application enables the comparison of various groups of interest as well as the incorporation of data from previous pilot studies, if available. In terms of operating systems, it runs on Windows. The PPCCA and DPPCA are applied to calculate sample size in high-dimensional data settings, the former being applied when there is no previous data by creating a simulated pilot data set, and the latter for the correlation in experimental pilot data. Finally, significance testing is based on two-sample t-statistics, while validation is based on a permutation approach used to estimate the null distribution of t-statistics discriminating against variables, accounting for correlation between metabolites and effect size variability, and accepting both nuclear magnetic resonance (NMR) and targeted mass spectrometry (MS) data.

Billoir et al. (2015) have developed an algorithm termed “Data-driven Sample size Determination” (DSD), based on MATLAB (Kernel and log-normal estimates) and GNU Octave (log-normal estimates) functions. It relies on orthogonal partial least squared (OPLS) analysis on Statistical Recoupling of Variables (SRV) clusters, with SRV being an automated variable size bucketing procedure aimed at identifying biological variables of interest, based on the statistical relationship between consecutive variables inherited from high-resolution bucketing (0.001 ppm wide buckets). SRV enables the selection of efficient recoupling parameters, like the typical singlet peak base width, the bucketing resolution, and the correlation threshold. The researcher can input different parameters, like the FDR controlled under negative correlation according to the Benjamini–Yekutieli correction. Unlike MetSizeR, DSD requires data from previous pilot studies and runs various operating systems, including Linux, Mac Os X, and Windows. Significance testing is based on the ANOVA, whilst validation is based on the “Receiver Operating Characteristic” (ROC) analysis and resampling under the null hypothesis. Moreover, Blaise et al. (2013, 2016, 2021) developed an algorithm in MATLAB that can be implemented to conduct a priori sample size determination and power analysis. This algorithm has been validated for data from humans and the model nematode organism *Caenorhabditis elegans*, obtained through NMR and liquid chromatography coupled with mass spectrometry (LC-MS). It consists of three steps: (i) modeling the distribution of pilot data, (ii) introduction of an artificial effect, and (iii) derivation of estimates and confidence intervals for performance metrics. More specifically, the algorithm relies on previous pilot studies that provide information used to manipulate and simulate large datasets, based on the complex, often non-linear relationship among sample size, statistical power, and effect size. Discrete or continuous outcomes and related effect sizes are computed, depending on the type of analysis (classification *versus* regression models, respectively). Subsets are created by random sampling from the original database, and various methods (univariate or multivariate) for effect detection are explored. Exploiting this novel framework, the authors demonstrated that a power of 0.8 can be achieved for some sample features with a 20-observation sample size, or that a cross-validated predictivity Q^2^_Y_ of 0.8 can be attained with 200 samples and an effect size of 0.2 (a small effect size). The Python version of this algorithm is termed PAPY and is a module of the PhenoMeNal (Peters et al., 2019), a comprehensive and standardized e-infrastructure for analyzing medical metabolic phenotype data (https://github.com/phnmnl/phenomenal-h2020/). Although the PhenoMeNal infrastructure has since been decommissioned, the methodological framework remains described in the literature.

Acharjee et al. (2020) have developed PowerTool, an application with a user-friendly, web-based interface (https://joelarkman.shinyapps.io/PowerTools/). The authors utilized both “artificial” (simulated) and “real-life” (previously published, or experimentally derived) data, to assess the performance of different state-of-the-art Random Forest (RF) algorithms, including the Boruta method, the permutation-based feature selection with and without correction methods, and the backward elimination-based feature selection method. The application performs ANOVA for the case of binary classification and linear regression tests for the case of continuous outcomes. Across a wide range of scenarios and input variable types, the Boruta methodology appeared to be the most stable technique, whereas, if allowed to stabilize over a number of iterations, the Permutation approach was able to offer the largest number of relevant features.

Li et al. (2020) have developed SSizer, an application for determining sample sufficiency in various biomedical fields, including metabolomics (https://idrblab.org/ssizer/). Appropriate sample size estimation is conducted based on multiple criteria, such as “statistical power” in terms of relevant and meaningful differences between comparative groups, “overall diagnostic and classification accuracies” based on cross-validation, and “robustness” of biomarker discoveries identified from several databases. SSizer has been validated on three randomly selected datasets obtained from the MetaboLights database, a freely available online repository for metabolomics studies. Unlike previously mentioned applications, SSizer supports a variety of machine learning techniques, such as Support Vector Machine (SVM), RF, and the “Diagonal Linear Discriminant Analysis” (DL-DA), among others. The user must input pilot data and the number of samples for the groups to be compared, even though SSizer can be used to estimate the power of studies involving not only two-group comparisons, but also multiple-group ones.

Wanichthanarak et al. (2017) have developed Metabox, an application written in R, with a web-based interface, running on Linux, Windows, and MAC OS X (http://kwanjeeraw.github.io/metabox/). Several pre-processing functions, including normalization methods, outlier detection, and data transformation, are performed by the application. The user must input meta-data, features, and quantified data (e.g., expression values). Power analysis is provided at the entity level for several tests, including Student’s t-test, Mann-Whitney U test, Kruskal-Wallis rank-sum test, ANOVA, repeated ANOVA, Friedman test, two-way ANOVA, two-way repeated ANOVA, and mixed ANOVA.

Finally, Liang et al. (2020) developed IP4M, an integrated, open-source, user-friendly platform for MS-based untargeted metabolomics data mining, as described in literature, but whose accessibility appears to be limited (https://ip4m.cn/, https://github.com/IP4/). It can handle data generated from both LC– and gas chromatography (GC)–MS. It enables data pre-processing and comprehensive data processing, performing several univariate (i.e., Student’s t-test, Wilcoxon test, ANOVA, and Kruskal-Wallis rank-sum test) and multivariate (i.e., PCA, PLS-DA, OPLS-DA, SVM, and RF) statistics. It runs on various operating systems, including Windows, Ubuntu, and macOS Catalina. It is written in Java, Perl, R, and Eclipse RCP.

In the field of integrative metabolomics, Syed et al. have developed MOPower (2021), an interactive R shiny application, available also as a code written in R and Python (https://hsyed.shinyapps.io/MOPower/, https://github.com/HSyed91/MOPower/). It enables the power computation for a dozen of integrative models, including (i) the joint regression modeling (jRM), such as the logistic and Cox proportional hazards regression, (ii) the mixed-effects joint regression modeling (MEjRM), (iii) the mediation analysis (MA), (iv) the multi-omics factor analysis (MOFA), (v) the sparse partial least square regression model with elastic net penalized regression to identify features, (vi) the joint non-negative matrix factorization (jNMF), (vii) the “Similarity Network Fusion” (SNF), (viii) the generalized linear modeling (GLM) and Cox Path with L1 penalty, (ix) the multi co-inertia analysis (MCIA), (x) the exact test, and xi) the Negative Binomial (NB) generalized log-linear regression model. The authors found that, for the specific study design model, power varied in parallel to the increase in feature number in a model-specific manner. For instance, MOFA showed an increase in power to detect a relevant association when the study sample size equally matched the number of features.

### Comparison

Taken together, these tools delineate three broad methodological orientations within the current resources for estimating statistical power and sample size in metabolomics and multi-omics research. The first orientation consists of simulation-based frameworks, exemplified by MetSizeR, DSD, PowerTools, SSizer and MOPower. Although different in their statistical architectures, these systems share a common goal to approximate, through synthetic data generation, the most relevant properties of the biological measurement process. Their reliance on simulated datasets makes it possible to explicitly examine how inferential performance changes under controlled manipulations of effect magnitudes, variance components, or feature correlations. This approach provides a more biologically and statistically realistic approximation, particularly when pilot data are available. However, its validity depends on the adequacy of modelling assumptions, including the representation of covariance structures, noise behavior, and the joint distribution of features. Among these simulation-based tools, SSizer stands out because it does not rely exclusively on statistical power as its criterion for determining sample sufficiency. Instead, it integrates three complementary forms of evidence: (i) statistical power estimated through the SSPA framework, (ii) diagnostic accuracy as quantified through cross-validated performance indicators such as the area under the receiver-operating characteristic curve, and (iii) robustness of discovered marker sets assessed through measures of reproducibility, such as overlap indices across repeated simulations. By assessing sample size adequacy simultaneously in terms of inferential sensitivity, predictive reliability, and feature-level stability, SSizer provides a multidimensional assessment of sufficiency that extends beyond conventional power calculations.

A second methodological orientation is embodied in effect-distribution–based analytical approaches, represented most clearly by metaX and by the POWER component within SSizer. Rather than producing artificial data, these methods derive power estimates from empirical distributions of effect sizes and variances extracted from the observed dataset. This strategy occupies an intermediate position between fully generative simulations and purely theoretical computations. It avoids the complexity of specifying high-dimensional generative models while retaining a principled link to the statistical structure of the underlying data. However, its inferential accuracy is intrinsically linked to the representativeness and quality of the empirical distribution. When the available dataset is limited or exhibits substantial noise, the estimated effect-size spectrum may offer an incomplete foundation for extrapolating power to alternative sample size regimes.

The third orientation consists of classical parametric power calculators embedded within broader metabolomics platforms such as MetaboAnalyst, IP4M and Metabox. These systems use analytical formulae derived from standard parametric tests, including t-tests, analysis of variance and linear regression. Their conceptual simplicity promotes accessibility and transparency and is often sufficient for targeted or moderately dimensional studies where assumptions of independence and homoscedasticity are reasonable approximations. However, because these frameworks do not incorporate extensive correlation structures characteristic of untargeted metabolomics and other high-dimensional omics modalities, they may produce sample size recommendations that are insufficiently sensitive to the multiplicity and covariance architecture inherent in discovery-driven analyses.

Across these three orientations, one observes a fundamental methodological spectrum in which the pursuit of biological realism must be balanced against computational tractability and interpretive clarity. Simulation-based approaches (first orientation) seek to preserve multivariate structure at the expense of greater complexity and model-dependence. Effect-distribution–based methods prioritize empirical grounding and computational efficiency but remain constrained by the quality of the underlying dataset, while classical parametric calculators offer interpretive simplicity but often abstract away the high-dimensional dependencies that shape the statistical behavior of omics data. The selection among these approaches therefore requires careful consideration of study design, the availability and reliability of pilot data, the complexity and correlations structure in the different layers of the omics data under investigation, and the degree of inferential nuance appropriate to the research objectives.

Although many researchers rely on conventional sample size calculators, typically based on univariate statistics such as t-tests combined with multiple-testing corrections, these approaches have important limitations when applied to metabolomics. Classical formulae implicitly assume independence among features, homoscedasticity, and low-dimensional settings, conditions that rarely are observed in highly correlated, multivariate metabolomics datasets. As a result, univariate power estimates often underestimate the effective degrees of freedom required, fail to account for covariance structures, and may yield overly optimistic sample size recommendations. In contrast, the dedicated tools reviewed here incorporate metabolomics-specific data characteristics: they model multivariate correlation patterns (MetSizeR, DSD), allow power estimation under high dimensionality or feature redundancy (SSizer, PowerTool), simulate and account for covariance structures and effect-size distributions, or evaluate downstream performance metrics such as classification accuracy, feature stability, and multivariate discrimination power. These capabilities provide a more biologically and statistically faithful assessment of required sample size than conventional one-metabolite-at-a-time approaches, especially in untargeted settings where discovery-driven analyses predominate.

### Practical applications

To assist researchers in selecting the most appropriate tool for a priori sample size calculation in metabolomics studies, differences in required inputs can support the selection of each tool. When no preliminary data from pilot studies are available, tools such as MetaSizerR and IP4M are recommended, as they are designed to operate under limited prior information. In cases where metabolomics biomarkers associated with the outcomes of interest are known, tools like PowerTool, SSizer, Metabox, metaX, and MOPower can be effectively employed for sample size estimation. Alternatively, the DSD and MetaboAnalyst tool can offer a valid alternative for estimating sample size.

When studies are based on population-level samples, additional challenges arise because these datasets typically exhibit higher inter-individual variability, and effects sizes tend to be smaller since they are influenced by multiple biological and environmental factors such as diet, lifestyle, genetics. To date, the number of tools considering metabolic phenotyping for population-based studies is very limited. While tools like MetaboAnalyst may serve to classify individuals into metabotypes, sample size estimation and power calculations for intervention studies, including, for instance, two metabotypes and both a treatment and control group (for a total of 4 arms), can be complicated. For example, SSizer may allow multiple-group comparison, while the lack of previous associations between metabolomics biomarkers and an outcome variable may limit the application of these tools. When limited information is available, more exploratory tools like MetaSizeR and IP4M should be regarded as valuable resources for metabotype-based controlled intervention studies. Regarding integrative metabolomics, the number of available tools is quite limited, with MOPower being the best choice. Further developments in this novel topic will surely expand the number of applications and methodologies available for more comprehensive metabolomics investigations.

In parallel with these methodological advances, the rapid evolution of artificial intelligence (AI) and, more specifically, generative AI paradigms are expected to further transform the landscape of sample size determination and power analysis in metabolomics. Emerging approaches based on deep generative models, including variational autoencoders and generative adversarial networks, offer the possibility to learn high-dimensional joint distributions of metabolomic features and to simulate realistic synthetic datasets that preserve complex covariance structures, non-linear dependencies, and latent biological variability. Such models may enable more accurate and flexible in silico experimentation, allowing researchers to explore a wide range of hypothetical study designs, effect sizes, and noise scenarios beyond the constraints of available pilot data. Moreover, large language models and AI-assisted analytical pipelines could facilitate automated model selection, parameter tuning, and adaptive study design, dynamically updating sample size requirements as new data become available. The integration of AI-driven causal inference frameworks and reinforcement learning strategies may further support optimal allocation of sampling resources under uncertainty, particularly in multi-omics and longitudinal settings. However, these opportunities must be balanced against challenges related to model interpretability, validation of synthetic data fidelity, and the risk of propagating biases embedded in training datasets. Future research should therefore focus on developing transparent, reproducible, and domain-informed AI methodologies that can be rigorously benchmarked against existing statistical frameworks, ultimately enabling a new generation of intelligent, data-adaptive power analysis tools.

An additional consideration relates to metabolomics studies conducted in the context of rare diseases or other scenarios where only limited sample sizes can be obtained. In such settings, the risks of reduced statistical power, inflated false-positive rates, and limited generalizability are particularly pronounced. Nevertheless, meaningful insights can still be extracted by adopting study designs and analytical strategies tailored to small cohorts. Approaches such as simulation-based power estimation (e.g., MetSizeR, DSD, SSizer), Bayesian modeling frameworks capable of stabilizing inference under sparse data, and the integration of prior biological knowledge or external datasets can help mitigate the constraints imposed by small sample sizes. Complementary strategies, such as data augmentation, synthetic data generation, or generative modeling (e.g., Bayesian simulators, GAN-based synthetic metabolite profiles), can further approximate realistic variance structures and enhance model training or validation when empirical data are scarce. Moreover, techniques that emphasize effect‐stability over strict significance, such as cross-validated feature selection, bootstrapping, or permutation-based validation, allow researchers to prioritize robustness even when classical power criteria cannot be fully met. Finally, collaborative multi-center data sharing and harmonized pre-analytical/analytical workflows offer a practical avenue to increase effective sample size while preserving the feasibility of studies involving rare conditions. Together, these strategies illustrate that, although limited sample sizes introduce inherent risks, rigorous design and modern computational tools enable metabolomics investigations to remain informative even under severe recruitment constraints. For further considerations, specifically related to sample size in the rare diseases’ context, works by Miller et al. (2018), Hee et al. (2017), Jackson and Jaki (2023), and Partington et al. (2022) are suggested.

## Conclusions and Perspectives

The number of samples required to identify statistically significant and clinically meaningful effects is a key aspect in biomedical studies, with implications for experimental designs, costs, and potential biomarker discoveries. Although several techniques for estimating appropriate sample size exist, this step remains challenging in the field of metabolomics. Moreover, the scarcity, or even the lack, of metabolomics pilot studies, as well as the assumptions that variables have equal variance and are independent of each other, make it challenging to use conventional tools to determine sample size in this field (Nyamundanda et al., 2013). As identified in this review, users can choose among different tools for sample size determination (i.e., MetaboAnalyst, MetSizeR, DSD, PowerTool, SSizer, Metabox, IP4M, metaX, and MOPower), whose selection could be mainly driven by available information, study design, and resources. Available options for intervention studies, including multiple-group comparisons, such as metabotype-based controlled trials, are limited. Only one study was identified regarding the application of sample size estimation and power analysis in the emerging subfield of integrative metabolomics was identified. Although significant progress has been made in the development of these tools, there is still room for improvement in terms of accessibility, ease of use, and the ability to function effectively without extensive prior data availability. Future developments should focus on creating more intuitive interfaces, expanding cross-platform support, and enhancing the ability to handle novel and diverse datasets. Moreover, given the importance of integrative metabolomics in providing valuable insights into the complexity of biological systems, aiding in understanding health/disease mechanisms, identifying diagnostic markers, and developing targeted therapies and preventive strategies, tools capable of handling this type of omics studies are recommended. In conclusion, efforts should be made to ensure that researchers have access to tools suited to their specific needs and expertise levels. The broad range of complexity levels and software dependencies highlights, indeed, a need for further development of more accessible and integrated tools that can cater for a broader user base, without compromising robustness and accuracy of the analyses. Given the importance of pre-determining appropriate sample sizes for discoveries and novel insights in the field of metabolomics, further methodological research is warranted.

## Supplementary Information

Below is the link to the electronic supplementary material.

Supplementary Material 1

## Data Availability

No datasets were generated or analysed during the current study.
