# Supplementary material for: A priori sample size determination and power analysis in metabolic phenotyping and integrative metabolomics: an application framework based on a systematic review of literature
Source: Metabolomics. 2026 Jun 19;22(4):104. doi: 10.1007/s11306-026-02464-y (PMC13282238; doi:10.1007/s11306-026-02464-y)
Supplement: Supplementary file 1 — Supplementary Material 1 [file 11306_2026_2464_MOESM1_ESM.docx]

**Supplementary Material**

***A priori* sample size determination and power analysis in metabolic phenotyping and integrative metabolomics: an application framework based on a systematic review of literature**

Nicola Luigi Bragazzi ^1^, Sara Dobani ^1*^, José Fernando Rinaldi de Alvarenga ^1^, Cristiana Mignogna ^1^, Daniele Del Rio ^1,2^, Pedro Mena ^1,2^

1. Human Nutrition Unit, Department of Food and Drug, University of Parma, Medical School, Building C, Via Volturno 39, 43125 Parma, Italy.

2. Microbiome Research Hub, University of Parma, Parco Area delle Scienze 27/A, 43124 Parma, Italy.

* **Correspondence to**: Human Nutrition Unit, Department of Food and Drug, University of Parma, Medical School, Building C, Via Volturno 39, 43125 Parma, Italy. E-mail address: [sara.dobani@unipr.it](mailto:sara.dobani@unipr.it)

**Supplementary Table 1.** Search strategy implemented in the present systematic review.

| **Search item** | **Details** |
| --- | --- |
| Search string MEDLINE *via* PubMed | ((((((((((("sample size calculation") OR ("sample size determination")) OR ("sample size analysis")) OR ("sample size estimation")) OR ("calculation of sample size")) OR ("determination of sample size")) OR ("sample sufficiency")) OR ("power analysis")) OR ("estimation of statistical power")) OR ("calculation of statistical power")) OR ("determination of statistical power")) AND (((((((((metabolomic*) OR (metabonomic*)) OR (metabotyp*)) OR ("metabolic phenotyp*")) OR ("metabolic biomarker*")) OR ("metabolic marker*")) OR ("metabolomics-based discovery")) OR ("metabonomics-based discovery")) OR ("metabolomic fingerprint*")) |
| Search string Scopus | ((TITLE-ABS-KEY ("metabolomic fingerprint*")) OR (TITLE-ABS-KEY ("metabonomics-based discovery")) OR (TITLE-ABS-KEY ("metabolomics-based discovery")) OR (TITLE-ABS-KEY ("metabolic marker*")) OR (TITLE-ABS-KEY ("metabolic biomarker*")) OR (TITLE-ABS-KEY ("metabolic phenotyp*")) OR (TITLE-ABS-KEY (metabotyp*)) OR (TITLE-ABS-KEY (metabonomic*)) OR (TITLE-ABS-KEY (metabolomic*))) AND ((TITLE-ABS-KEY ("determination of statistical power")) OR (TITLE-ABS-KEY ("calculation of statistical power")) OR (TITLE-ABS-KEY ("estimation of statistical power")) OR (TITLE-ABS-KEY ("power analysis")) OR (TITLE-ABS-KEY ("sample sufficiency")) OR (TITLE-ABS-KEY ("determination of sample size")) OR (TITLE-ABS-KEY ("calculation of sample size")) OR (TITLE-ABS-KEY ("sample size estimation")) OR (TITLE-ABS-KEY ("sample size analysis")) OR (TITLE-ABS-KEY ("sample size determination")) OR (TITLE-ABS-KEY ("sample size calculation"))) |
| Time filter | None applied (from inception) |
| Language filter | None applied |
| Target journals hand-searched | Anal Chem; BMC Bioinformatics; BMC Med Genomics; Brief Bioinform; J Mol Biol; Metabolomics, Nucleic Acids Res; Stat Med |

**Supplementary Methods. Study protocol for *a priori* sample size determination and power analysis in metabolic phenotyping and integrative metabolomics**.

*Abstract*

The review aims to provide a comprehensive framework for conducting *a priori* sample size determination and power analysis in metabolic phenotyping and integrative metabolomics studies. Given the complexity and high dimensionality of metabolomics data, the review will detail the methodologies and tools available, addressing the challenges and proposing best practices for accurate and reliable sample size calculation.

*Introduction*

Detection of relevant and clinically meaningful biomedical effects in metabolomics studies relies heavily on correctly determining the number of samples and observations before starting the study. This is critical in metabolic phenotyping, where the comprehensive analysis of metabolites in biological samples provides insights into the metabolic profiles of organisms. Despite advancements, sample size determination in metabolomics remains a challenging step due to the high chemical and physical diversity of metabolites, the complexity, and variability of the data. Integration of metabolomics data with other omics technologies deserves further investigation.

*Objectives*

The objectives of the review are:

- to systematically review the literature on *a priori* sample size determination and power analysis methods in metabolomics;
- to identify and evaluate the available tools and methodologies for sample size calculation;
- and to propose a practical framework for selecting and applying these tools in metabolomics studies;
- discuss the tools available for metabotype-based intervention studies and integrative metabolomics.

*Methods*

A comprehensive literature search will be carried out by mining two major electronic, scholarly databases (PubMed/MEDLINE and Scopus).

The search strategy will imply using keywords such as “sample size calculation”, “sample size determination”, “power analysis”, and “metabolomics”, combined with Boolean operators and Medical Subject Headings (MeSH) terms. Studies providing methodological insights on sample size estimation and power analysis in metabolomics will be included, while theoretical/mathematical papers without practical applications or software validation will be excluded.

The following parameters will be extracted: study authors, application name, programming language, operating systems, requirement of pilot studies, data types, pre-processing steps, user input parameters, and types of statistical tests.

Two independent researchers will extract these data using an ad hoc Excel spreadsheet, with disagreements resolved by consensus. During the data synthesis step, the findings will be summarized in a narrative manner, supplemented with tables and charts to illustrate the characteristics of the identified tools. A comparative analysis will be conducted to evaluate the strengths and limitations of each tool based on criteria such as ease of use, versatility, and the requirement for pilot data.

*Results*

The expected outcomes are:

- the identification of tools and methodologies for sample size determination in metabolomics, along with their application scenarios and limitations;
- the common challenges faced in sample size determination for metabolomics (such as high-dimensionality, biological variability, and lack of pilot data) will be discussed;
- researchers will be provided with recommended best practices, guidelines, and checklists, including the use of specific tools based on study design and data availability;
- specific aspects related to metabolic phenotyping and integrative metabolomics will be discussed;
- this review will identify potential gaps in the field.

*Conclusion*

The review will guide researchers in selecting and applying the appropriate tools for sample size determination and power analysis in metabolomics studies, ultimately improving the reliability and reproducibility of their findings.
